# Radiotherapy combined with docetaxel alters the immune phenotype of HNSCC cells and results in increased surface expression of CD137 and release of HMGB1 of specifically HPV-positive tumor cells

**DOI:** 10.1016/j.neo.2023.100944

**Published:** 2023-10-17

**Authors:** Fridolin Grottker, Simon Gehre, Clara M. Reichardt, Azzaya Sengedorj, Tina Jost, Thorsten Rieckmann, Markus Hecht, Antoniu-Oreste Gostian, Benjamin Frey, Rainer Fietkau, Udo S. Gaipl, Michael Rückert

**Affiliations:** a Translational Radiobiology, Department of Radiation Oncology, Universitätsklinikum Erlangen, Friedrich-Alexander-Universität Erlangen-Nürnberg, Germany; b Department of Radiation Oncology, Universitätsklinikum Erlangen, Friedrich-Alexander-Universität Erlangen-Nürnberg, Germany; c Comprehensive Cancer Center Erlangen-EMN, Erlangen, Germany; d Laboratory of Radiobiology & Experimental Radiation Oncology, University Medical Center Hamburg-Eppendorf, Hamburg, Germany; e Department of Otorhinolaryngology, University Medical Center Hamburg Eppendorf, Germany; f Department of Radiotherapy and Radiation Oncology, Saarland University Medical Center, Homburg, Germany; g Department of Otorhinolaryngology, Universitätsklinikum Erlangen, Friedrich-Alexander-Universität Erlangen-Nürnberg, Germany; h Clinic for Otorhinolaryngology, Head and Neck Surgery and Facial Plastic Surgery, Klinikum Straubing, Germany

**Keywords:** Radiotherapy, Docetaxel, Head and neck squamous cell carcinoma, HPV status, Immune checkpoint molecules, HMGB1, Dendritic cells, Anti-tumor immunity

## Abstract

**Purpose:**

Human papilloma virus (HPV) positive head and neck squamous cell carcinoma (HNSCC) tumors respond significantly better to anticancer treatments. It is assumed to be due to a better response to radiotherapy (RT), and presumably to an increased immunogenicity. However, little is known how the immune phenotype of HNSCC tumor cells is modulated by standard treatment, namely by radiochemotherapy (RCT).

**Methods:**

Therefore, we aimed to examine the impact of the HPV status on the immune phenotype of HNSCC cell lines following RCT with 5 × 3Gy or 1 × 19.3Gy and/or docetaxel, by analyzing cell death, release of damage-associated molecular patterns (DAMPs), surface expression of immune checkpoint molecules (ICMs) and the impact on activation of human monocyte-derived dendritic cells (hmDCs).

**Results:**

Cell death induction and Hsp70 release following RCT was independent of the HPV status, and RCT significantly increased the expression of the immune suppressive ICMs PD-L1, PD-L2 and HVEM. An immune stimulatory ICM, CD137, was significantly increased following RCT only on HPV-positive cell lines, as well as the release of HMGB1. Although the treatment increased cell death and modulated ICM expression in HNSCC, the hmDCs were not activated after co-incubation with treated tumor cells.

**Conclusion:**

Our data with the HPV-dependent release of HMGB1 and increased expression of CD137 following RCT provide a hint for increased immunogenicity underlining the better prognosis for HPV positive tumors following RCT.

## Highlights

• RCT-treated HPV-positive HNSCC tumors are associated with the release of HMGB1 and an increased expression of CD137.
• This highlights a potential mechanism for the better prognosis for HPV-positive tumors following RCT.
• ICMs other than PD-1/PD-L1, such as HVEM, were identified that could be used as a potential target in multimodal therapy approaches for HNSCC in the future.

## Introduction

Head and neck squamous cell carcinomas (HNSCC) are the most common malignancies that arise in the head and neck region. They develop from the mucosal epithelium in the oral cavity, pharynx and larynx [1]. The main risk factors of HNSCC are alcohol and tobacco abuse, but also an infection with the human papilloma virus (HPV). The conventional therapies consist of surgery and radiotherapy (RT). In more advanced tumor stages, RT is complemented by chemotherapy (CT) using either cisplatin, fluorouracil or docetaxel [2,3].

RT is mostly used with classical fractionation schemes with a single dose per fraction of 2 Gy, e.g. for head and neck cancers 68-70 Gy in 34-35 fractions. However, due to improved radiation techniques such as stereotactic body RT (SBRT), the Covid19 pandemic aiming for reduced visits at the hospitals, and the expanding knowledge of immune activation by hypofractionated RT, single doses higher than 2 Gy in hypofractionated protocols (higher single dose per fraction and less total fractions) are increasingly tested and used in the clinics [4]. We therefore used a single dose per fraction of 3 Gy in our model system and additionally mimicked the clinical situation by repeated irradiation (5 × 3 Gy). For comparison, a single high dose was used. RT induces DNA damage in tumor cells, which, if beyond the cells repair mechanisms, will lead to cell cycle arrest or ideally in tumor cell death. However, irradiation is also known for its immune-modulatory effects, e.g. resulting in immunogenic cell death (ICD), for example in the form of necrosis and/or by the release of different damage-associated molecular patterns (DAMPs), such as heat shock protein 70 kDa (HSP70), ATP and high-mobility group protein 1 (HMGB1) [5], [6], [7]. However, even tumor cell apoptosis can be immunogenic, e.g. when the dying cells express calreticulin on their surface [8].

As proven for RT, also certain chemotherapeutic agents (CTA) bear immune modulatory potential, mostly based on inducting of ICD. The resulting immune modulatory efficacy of conventional chemotherapeutic agents including taxanes has been shown to be much higher in immune competent mouse models than in their immune deficient counterparts [9]. Docetaxel, being a CTA that was proven to enhance overall survival of HNSCC patients in multimodal settings [3], belongs to the group of taxanes with high immune stimulatory potential [10]. However, it is not known how combination of docetaxel with RT impact on the immune phenotype of HNSCC tumor cells, while the immunogenic potential of RT and cisplatin in human tumor models of HPV-associated malignancies was just recently analyzed [11].

It has been generally suggested that HPV positive HNSCC are more immunogenic compared to HPV negative ones [12]. We have just recently identified that following RT, the expression of the inducible co-stimulatory molecule ligand (ICOS-L) is upregulated only on HPV positive HNSCC cell lines [13]. As ICOS/ICOSL signaling leads to the activation, proliferation and survival of cytotoxic T cells [14], it might foster RT-induced anti-HNSCC tumor responses. In general, HPV positive HNSCC tumors have a better prognosis. The overall survival (OS) of patients was shown to be significantly higher compared to HPV negative tumors [15,16]. HPV positive HNSCC tumors are more sensitive to RT and RCT [17], [18], [19], but Rieckmann et al. indicated that the group of HPV positive HNSCC tumors per se are quite heterogenic in their radiosensitivity, which means that they can be also less radiosensitive than other HPV negative cell lines [20].

We hypothesized that RT in combination with docetaxel, besides inducing cancer cell death, impacts the expression of several immune checkpoint molecules on HNSCC in dependence of the HPV status and additionally the release of certain DAMPs. This might result in activation of dendritic cells (DCs). The latter ideally capture antigens released by the tumor cell, cross-present them and finally thereby prime and activate T cells. Here, the presence of co-stimulatory signals like CD80, CD86, CD70, CD137, OC40 and stimulatory immune checkpoint molecules (ICM) are pivotal [21]. However, cancer cells often can evade the immune system [22]. One way of tumor cells repressing an effective immune response, especially the T cell response, is via expression of immune suppressive ICMs [23].

To test our hypothesis, we here treated two HPV positive and two HPV negative HNSCC cell lines with RT, docetaxel or a combination of both and consecutively analyzed tumor cell death forms, release of the DAMPs HSP70 and HMGB1, the surface expression of several ICMs and finally the activation of DCs by the treated tumor cells.

## Material and methods

### Cell lines and cell culture

Four human HNSCC cell lines (HPV positive: UM-SCC-47, UD-SCC-2 and HPV negative: HSC4, Cal33) were examined. All four cell lines were cultivated in Dulbecco's modified Eagle's medium (DMEM, Pan-Biotech GmbH, Aidenbach, Germany) supplemented with 10 % fetal bovine serum (FBS, Biochrom AG, Berlin, Germany) and 1 % Penicillin-Streptomycin (PenStrep, Gibco, Carlsbad, CA, USA). The cell lines were passaged twice per week with Trypsin (Gibco Life Technologies, Carlsbad, CA, USA) for a maximum of 15 passages. All cell lines were tested negative for mycoplasma contamination. Peripheral blood mononuclear cells (PBMCs) derived from healthy human donors were cultured in “DC medium” consisting of RPMI-1640 (Merck, Darmstadt, Germany) supplemented with 1 % Pen/Strep, 1 % L-Glutamine (Gibco, Carlsbad, CA, USA), 1 % Hepes buffer (Gibco Life Technologies, Waltham, MA, USA) and 1 % human heat inactivated serum (Gibco, Carlsbad, CA, USA).

All cells were cultivated in standard conditions (37 °C, 5 % CO_2_ and 95 % humidity).

### Treatments and sampling

**Fig. 1:**
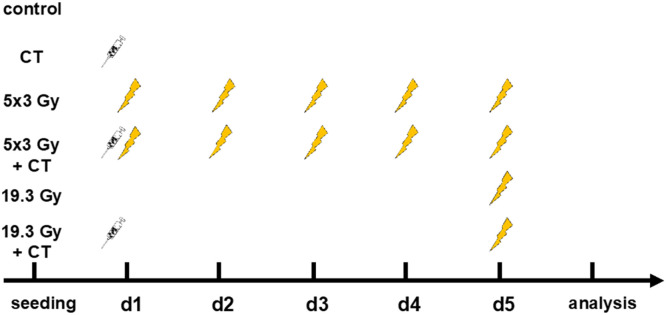
Treatment scheme. The cells for the different treatment approaches were seeded 24 h before d1. On d1 0.5 nM of docetaxel was added to the group CT, 5 × 3 Gy + CT and 19.3 Gy + CT. 5 × 3 Gy and 5 × 3 Gy + CT were irradiated with 3 Gy on d1-d5. 19.3 Gy and 19.3 Gy + CT were irradiated with 19.3 Gy on d5. 24 h after the last treatment cells were harvested for flow cytometry.

The cells were irradiated using an X-ray tube (X-Ray tube Isovolt Titan, GE Sensing & Inspection, Boston, USA) in a lead shielding chamber. The 3 Gy therapy group was irradiated on d1 – d5, the 19.3 Gy therapy group on d5. The taxane docetaxel was added on d1 in a final concentration of 0.5 nM. The cells were collected, as well as supernatant taken for Hsp70 and HMGB1 ELISA analyses, 24 h after the last irradiation (Fig. 1).

### Cell death analyses

0.1 × 10^6^ cells were stained with 100 μl of cell death staining solution (1 ml of Ringer's solution (Fresenius Kabi, Bad Homburg, Germany), 0.75 μl/ml of AnnexinV-FITC (AxV) (1 mg/ml; GeneArt, Regensburg, Germany), and 1.0 μl/ml of Propidium iodide (Pi) (1 mg/ml; Sigma-Aldrich, Munich, Germany). After incubation for 30 min at 4 °C in the dark, the measurement was performed on a CytoFLEX S flow cytometer (Beckman Coulter, Brea, CA, USA) and analyzed with the Kaluza Analysis software (Beckman Coulter, Brea, CA, USA).

### Immune checkpoint molecule expression analysis

0.1 × 10^6^ cells were stained with staining solution containing FACS buffer (PBS, Sigma-Aldrich, Munich, Germany), 2 % FBS and 2 mM EDTA (Carl Roth, Karlsruhe, Germany) and Zombie NIR (live/dead) alone or Zombie NIR and antibodies (Table 1). Before analyzing the cells with the CytoFLEX S flow cytometer, they were incubated for 30 min at 4 °C in the dark. The ∆MFI (mean fluorescence intensity) for every ICM was calculated by subtracting the MFI of the Zombie-only-stained sample from the MFI of the Zombie-and-antibody stained one, to correct for treatment-related autofluorescence.

**Table 1:** List of flow cytometry antibodies and dyes for immune checkpoint molecule analysis on HNSCC cells.

**Target antigen**	**Fluorochrome**	**Product number**	**Supplier**	
CD70	FITC	355106	BioLegend	San Diego, CA, USA
HVEM/CD 270	APC	318808	BioLegend	San Diego, CA, USA
ICOS-L/CD 275	BV 421	564276	BD Bioscience	New Jersey, NJ, USA
OX40L/CD 134	PE	326308	BioLegend	San Diego, CA, USA
PD-L1/CD 274	BV 605	329724	BioLegend	San Diego, CA, USA
PD-L2/CD 273	APC	345508	BioLegend	San Diego, CA, USA
TNFRSF9/CD 137	BV 421	311508	BioLegend	San Diego, CA, USA
Live/dead	Zombie NIR	423105	BioLegend	San Diego, CA, USA

### Protein analysis with Enzyme Linked Immunosorbent Assay (ELISA)

The concentration of HMGB1 and HSP70 in the treated head and neck cancer cell supernatant was analyzed with sandwich ELISA assays. The assays were performed according to the manufacturer's recommendations of the HSP70 ELISA kit (Human/Mouse/Rat Total HSP70/HSPA1A ELISA, R&D Systems, Minneapolis, MN, USA) and the HMGB1 ELISA kit (Ibl International). The absorbance at 450 nm (HMGB1, HSP70), 540 nm (HSP70), as well as 650 nm (HMGB1) was read using the Epoch Microplate Spectrometer.

### Generation of monocyte-derived dendritic cells (hmDCs) from peripheral blood mononuclear cells (PBMC)

Human peripheral blood mononuclear cells (PBMCs) were isolated from leukoreduction system chambers (LRSC) of healthy human donors using density gradient centrifugation in SepMate^TM^ PBMC Isolation Tubes (Stemcell, Vancouver, Canada) and Lymphoflot (Biotest AG, Dreieich, Germany). Then, they were washed twice at 4 °C with PBS (Sigma-Aldrich, Munich, Germany)/0.5 mM EDTA (Carl Roth, Karlsruhe, Germany) and RMPI-1640. In the following, 30 × 10^6^ cells each were seeded on cell culture dishes, which were IgG-pre-coated, in 10 ml of DC medium and incubated for 1 h at 37 °C. Then, all the non-attached cells were removed by rinsing the dishes with fresh DC medium. Finally, 10 ml of fresh DC medium was added.

**Fig. 2:**
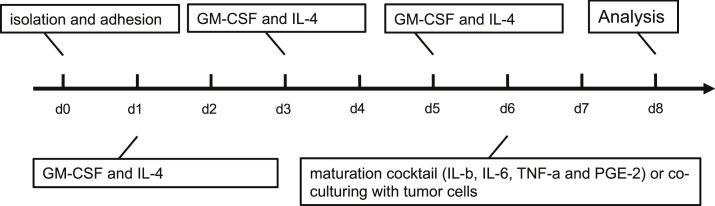
Generation of monocyte-derived dendritic cells (hmDCS). The latter were differentiated from peripheral blood mononuclear cells (PBMCs) for 5 days before they were co-cultured with untreated and treated HNSCC cells.

24 h after seeding, the old DC medium was removed again and 10 ml of RPMI containing 800 U/ml (0.57 μl/ml) of GM-CSF (Miltenyi Biotec, Bergisch Gladbach, Germany) and 500 U/ml (5 μl/ml) of IL-4 (ImmunoTools, Friesoythe, Germany) were added to each cell culture dish. On day 3, 4 ml of RPMI and 800 U/ml (0.57 μl/ml) of GM-CSF and 500 U/ml (5 μl/ml) of IL-4 were added. On day 5, 4 ml of RPMI with half of the earlier used amount of GM-CSF (400 U/ml = 0.285 μl/ml) and IL-4 (250 U/ml = 2.5 μl/ml) were added (Fig. 2).

### Maturation induction and co-culture of the hmDCs with treated tumor cells

On day 6 of the DC differentiation the immature DCs were harvested using a serological pipette. Afterwards, treated tumor cells with tumor medium were seeded together with immature DCs with DC medium in a 2:1 ratio (tumor cells: DCs) in 6 well plates. As a positive control, DCs were treated with a maturation cocktail (MC) containing 13.16 ng/ml of IL-1β (ImmunoTools, Friesoythe, Germany), 1000 U/ml of IL-6 (ImmunoTools, Friesoythe, Germany), 10 ng/ml of TNF-α (ImmunoTools, Friesoythe, Germany) and 1 μg/ml of PGE-2 (Pfizer, Berlin, Germany). 48 h after the co-incubation, the cells were harvested mechanically using a cell scraper. Half of the cells was stained with a live/dead stain only, the other half with a staining solution additionally containing antibodies for different DC activation markers (see Table 2). Using multicolor flow cytometry, the MFI of the respective markers was measured at a CytoFLEX S flow cytometer. ∆MFI of the corresponding cells was calculated by deducting the MFIs of the live/dead-only from the full staining.

**Table 2:** List of flow cytometry antibodies and dyes for DC maturation analysis.

**Target antigen**	**Fluorochrome**	**Product number**	**Supplier**	
CD70	FITC	355106	BioLegend	San Diego, CA, USA
CD80	PE-Cy 7	305218	BioLegend	San Diego, CA, USA
CD83	PE	556855	BD Pharmingen	Franklin Lakes, NJ, USA
CD86	BV	305428	BioLegend	San Diego, CA, USA
HLA-DR	APC-Vio 770	130-123-550	Miltenyi Biotech	Bergisch Gladbach, NRW, GER
Live/dead	Zombie yellow	423104	BioLegend	San Diego, CA, USA

## Results

### Radiotherapy combined with docetaxel induces apoptosis and necrosis of HNSCC tumor cells independently of the HPV status

**Fig. 3:**
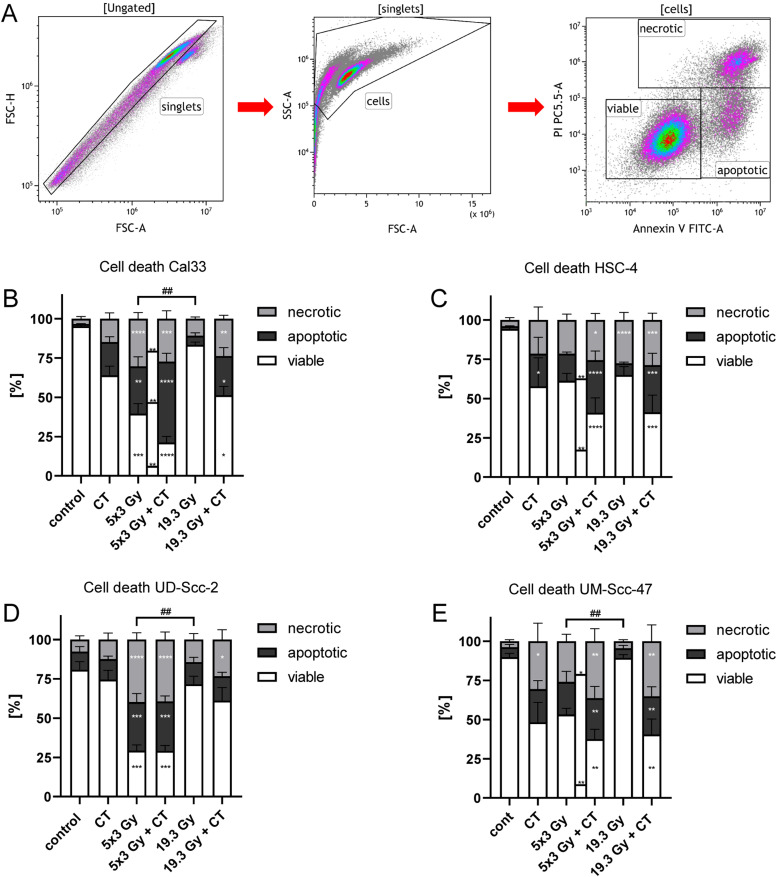
Combined radiochemotherapy results in the highest cell death rates of HNSCC cells independent of the HPV status. A: After pre-gating on singlets and then excluding the debris, the remaining cells were identified as viable (AnnexinV-, PI-), apoptotic (AnnexinV+, PI-), or necrotic (AnnexinV+, PI+). B-E: Cell death analyses of the HPV-negative cell lines Cal33 (B) and HSC-4 (C) and the HPV-positive cell lines UD-Scc-2 (D) and UM-Scc-47 (E) are shown as stacked bars showing the mean ±SD. A Kruskal-Wallis test with Dunn's correction for multiple testing was calculated to compare the different treatment approaches versus the control. Furthermore, a Mann-Whitney test was calculated to compare 5 × 3 Gy and 5 × 3 Gy + CT (**), as well as 5 × 3 Gy and 19.3 Gy (##); *p < 0.05, **p < 0.01, ***p < 0.001, ****p < 0.0001; ##*p* < 0.01 *n* = 6.

We first analyzed cell death induction and cell death forms following the different treatment approaches (Fig. 1) for all four HNSCC cell lines. The gating strategy for detection of viable, apoptotic and necrotic tumor cells is depicted in Fig. 3A. Exclusive docetaxel treatment caused a significantly increased apoptotic rate for HSC-4 cells and necrotic rate for UM-Scc-47 cells (Fig. 3C, E). Hypo-fractionated irradiation significantly augmented both, apoptosis and necrosis in Cal33 and UD-Scc-2 (Fig. 3B, D), whereas no significant differences occurred in the other cell lines. In all but the HSC-4 cell line, the percentage of viable cells was significantly reduced after the 5 × 3 Gy irradiation scheme.

The combination of RT with 5 × 3 Gy and CT with docetaxel lead to a significantly reduced viability of the cells and to a higher apoptotic and necrotic rate in all cell lines. Again, in all cell lines, the single high irradiation dose of 19.3Gy combined with CT significantly increased necrosis and lead to significantly higher percentage of apoptosis in all cell lines but UD-Scc-2 (Fig. 3B-E). Only RT with a single dose of 19.3 augmented the necrosis rate only in the HPV-negative cell line HSC-4 (Fig. 3C).

### Release of HMGB1 following hypo-fractionated RT in combination with docetaxel is associated with the HPV status

**Fig. 4:**
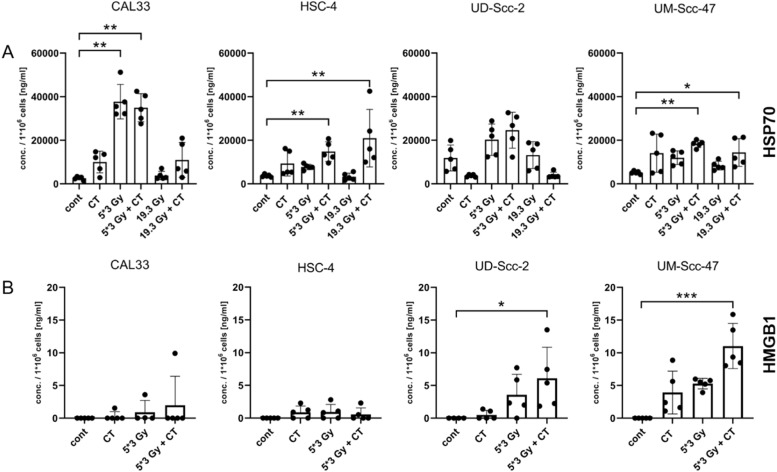
Treatment-dependent release of HSP70 and treatment- and HPV-dependent release of HMGB1 of HNSCC cells. The supernatants of the tumor cells were collected 24 h after the last treatment for ELISA analyses of HSP70 (A) and HMGB1 (B). The concentrations in ng/ml per 1*10^6^ cells are shown as stacked bars showing the mean with ±SD. A Kruskal-Wallis test with Dunn's correction for multiple testing was calculated to compare the different treatment approaches versus the control. Non-measurable values were set to 0; *p < 0.05, **p < 0.01, ***p < 0.001, n ≥ 4.

Next, the release of the DAMPs HSP70 and HMGB1 following RCT was analyzed. HSP70 was found in significantly higher amount in supernatants of the cell lines Cal33, HSC-4 and UM-Scc-47 after hypo-fractionated irradiation in combination with docetaxel (Fig. 4A). A single high dose irradiation plus CT only led to a significant increased release of HSP70 for the cell line HSC-4 and UM-Scc-47 (Fig. 4A). No significant changes could be detected regarding UD-Scc-2, however a tendency of an increase particularly after 5 × 3Gy plus docetaxel is observed (Fig. 4A). Both docetaxel and the single high irradiation dose had no significant impact on HSP70 release.

Regarding the DAMP HMGB1, both HPV-positive cell lines UD-Scc-2 and UM-Scc-47 had a significantly increased concentration of HMGB1 in their supernatant following hypo-fractionated RT plus docetaxel. In contrast, no significant release was detected for the HPV-negative cell lines Cal33 and HSC-4 (Fig. 4B).

### Treatment-dependent upregulation of stimulatory immune checkpoint molecules is found especially in HPV-positive HNSCC cells

**Fig. 5:**
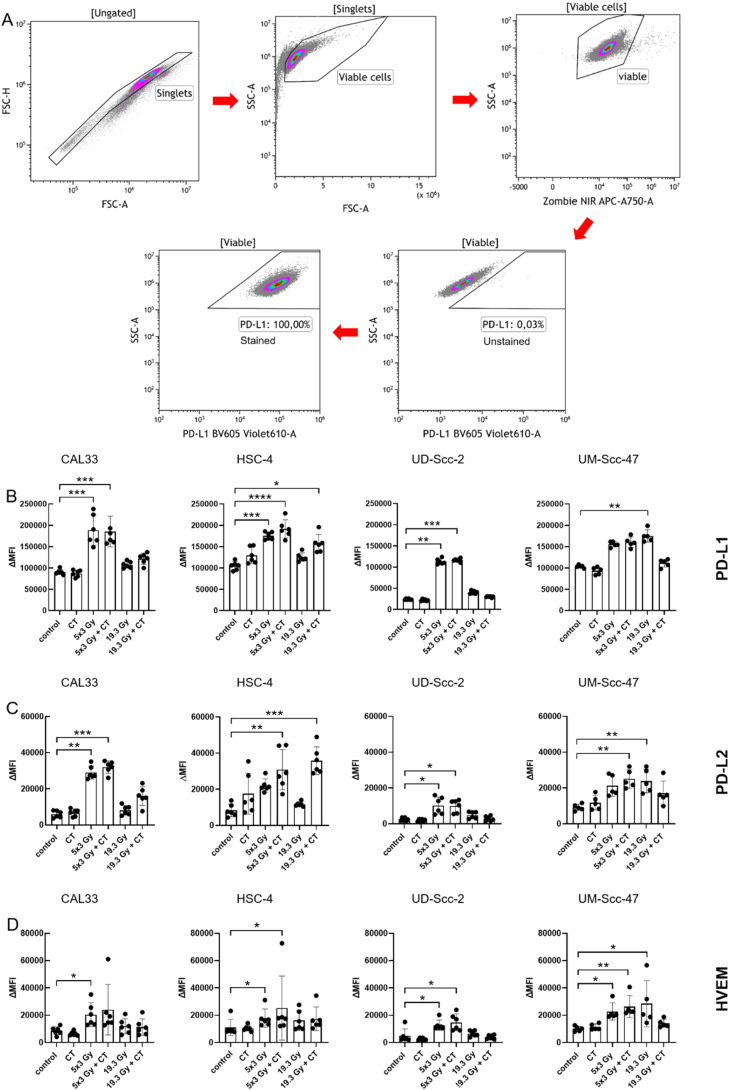
Hypo-fractionated radiotherapy alone and in combination with docetaxel increases the expression of inhibitory checkpoint molecules independently of the HPV-status of HNSCC cells. 24 h after the last treatment cells were harvested for flow cytometry. A: After pre-gating on the singlets, debris was excluded. The viable cells were detected via the Zombie NIR viable/dead stain. Immune checkpoint molecule (ICM) expression is presented in the graphs as ∆MFI (mean fluorescence intensity) which was calculated by subtracting the MFI of the Zombie-only-stained samples from the respective Zombie-and-antibody-stained samples. B-D: For the HPV-negative cell lines Cal33 and HSC-4, as well as HPV-positive cell lines UD-Scc-2 and UM-Scc-47, the mean fluorescence intensity (ΔMFI) of the immune suppressive checkpoint molecules PD-L1 (B), PD-L2 (C), HVEM (D) are shown as stacked bars showing the mean with ±SD. A Kruskal-Wallis test with Dunn's correction for multiple testing was calculated to compare the different treatment approaches versus the control. *p < 0.05, **p < 0.01, ***p < 0.001; n≥5.

As tumor cells can also modulate the anti-tumor immune response in direct contact to immune cells, we next analyzed the surface expression of stimulatory and suppressive immune checkpoint molecules (ICMs) on tumor cells following the different treatments and an exemplarily analysis for PD-L1 is shown in Fig. 5A. It is of note that docetaxel treatment alone had no impact on the surface expression of the analyzed suppressive ICMs.

However, predominantly hypo-fractionated radiotherapy alone or in combination with docetaxel resulted in a significant increase for almost all analyzed inhibitory immune checkpoint molecules in all four cell lines (Fig. 5B-D). Single irradiation with 19.3 Gy had only an effect on the UM-Scc-47 cell line and significantly upregulated PD-L1, PD-L2 and HVEM expression. In combination with chemotherapy, however, their expression was not affected anymore. 19.3 Gy plus CT significantly increased only the expression of PD-L1 and PD-L2 in the HSC-4 cell line.

**Fig. 6:**
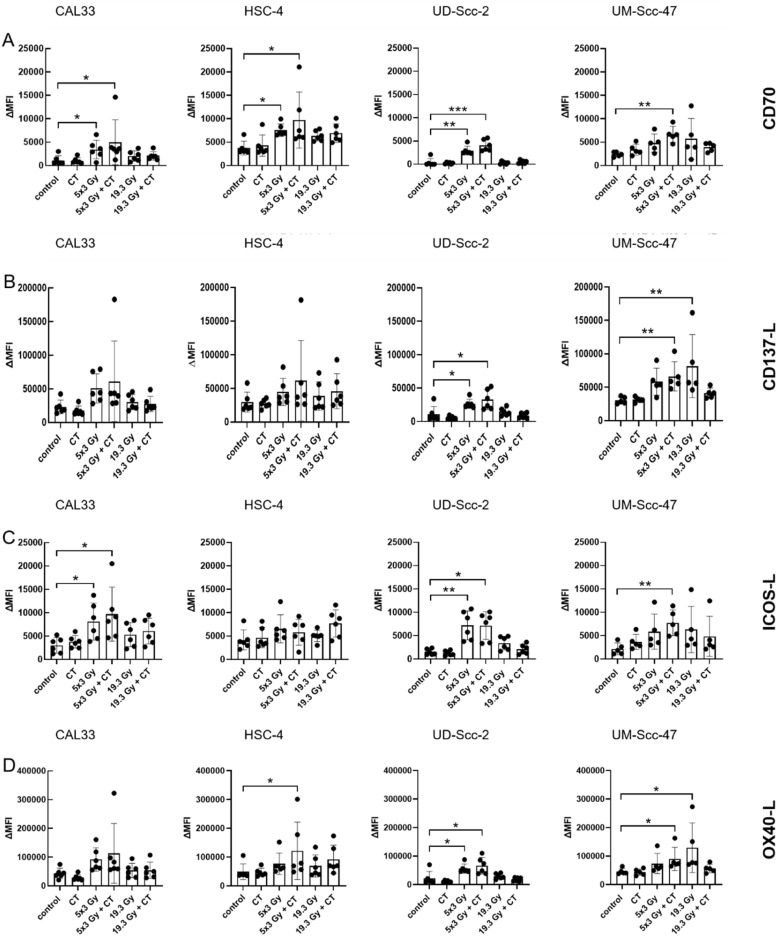
HPV-status of HNSCC cells impacts on expression of stimulatory immune checkpoint molecules. 24 h after the last treatment cells were harvested for flow cytometry. After pre-gating on the singlets, debris was excluded. The viable cells were detected via the Zombie NIR viable/dead stain. Immune checkpoint molecule (ICM) expression is presented in the graphs as ∆MFI (mean fluorescence intensity) which was calculated by subtracting the MFI of the Zombie-only-stained samples from the respective Zombie-and-antibody-stained samples. A-D: For the HPV-negative cell lines Cal33 and HSC-4, as well as HPV-positive cell lines UD-Scc-2 and UM-Scc-47, the mean fluorescence intensity (ΔMFI) of the immune suppressive checkpoint molecules CD70 (A), CD137-L (B), ICOS-L (C) and OX40-L (D) are shown as stacked bars showing the mean with ±SD. A Kruskal-Wallis test with Dunn's correction for multiple testing was calculated to compare the different treatment approaches versus the control. *p < 0.05, **p < 0.01, ***p < 0.001; n≥5.

Further, we also analyzed the immune stimulatory immune checkpoint molecules CD70, CD137-L, ICOS-L and OX40-L on the tumor cell surface. Here, hypo-fractionated RT in combination with docetaxel significantly increased the expression of all stimulatory immune checkpoint molecules in both HPV-positive cell lines. In contrast, only CD70 and ICOS-L was significantly augmented for the cell line Cal33, CD70 and OX40-L for HSC-4, which are both HPV-negative (Fig. 6). An increased surface expression of CD137 following RCT with 5 × 3 Gy was only observed on the HPV-positive tumor cells. Exclusive chemotherapy did not cause any significant changes.

A single high dose of RT resulted in a significant increase of the stimulatory immune checkpoint molecules CD137-L and OX40-L regarding the cell line UM-Scc-47, whereas the other cell lines did not show any significant changes and also additional chemotherapy did not modulate their surface expression (Fig. 6).

### The co-incubation of treated HPV-positive and HPV-negative tumor cells had no significant effect on the expression of activation markers on hmDCs

**Fig. 7:**
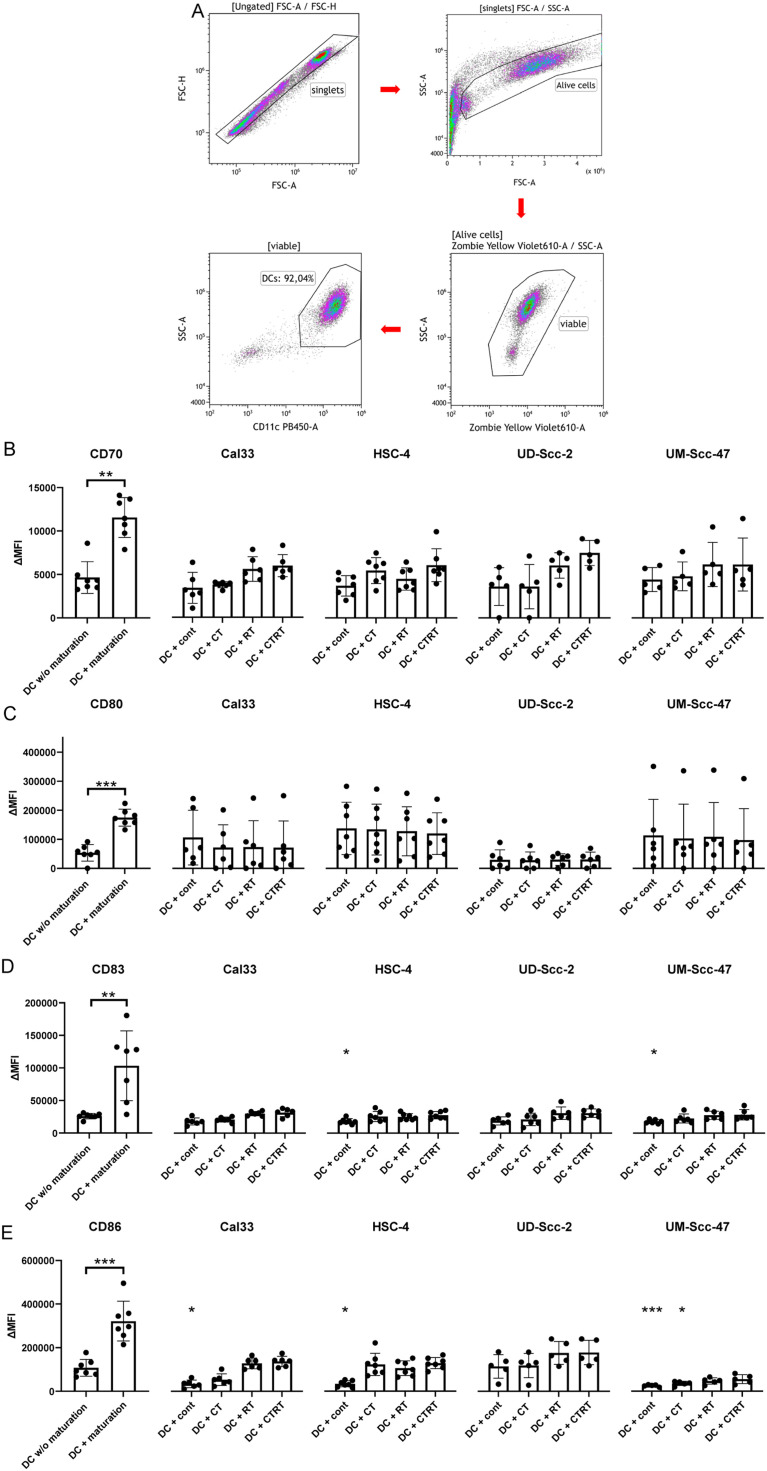
Co-incubation of treated HPV-positive and HPV-negative HNSCC tumor cells had no significant effect on the expression of activation markers of hmDCs. 24 h after the last treatment, the cells were harvested and co-cultured with hmDCs. 48 h after the co-incubation the hmDCs were analyzed using flow cytometry. A: Presenting the gating strategy. B-E: For the hmDCs, which were co-cultured with the HPV-negative cell lines Cal33 and HSC-4, as well as HPV-positive cell lines UD-Scc-2 and UM-Scc-47, the mean fluorescence intensity (ΔMFI) of CD70 (B), CD80 (C), CD83 (D) and CD86 (E) is shown as stacked bars showing the mean with ±SD. A Mann-Whitney test was calculated to compare hmDCs without maturation cocktail and hmDCs with maturation cocktail. Furthermore, a Kruskal-Wallis test with Dunn's correction for multiple testing was calculated to compare the different treatment approaches versus hmDCs without maturation cocktail. *p < 0.05, **p < 0.01, ***p < 0.001; n≥5.

To further characterize the immunogenicity of the treated HPV-negative and -positive tumor cells, we analyzed the activation of hmDCs co-cultured with tumor cells and their supernatant (Fig. 7). Only the use of a maturation cocktail led to a significant upregulation of CD70, CD80, CD83 and CD86 on hmDCs. Co-incubating the treated tumor cells of all cell lines with the hmDCs had no significant impact on the expression of all analyzed activation makers (Fig. 7B-E). In contrast, co-culturing untreated tumor cells with the hmDCs even significantly decreased the expression of CD83 for the cell line HSC-4 and UM-Scc-47 as well as the activation marker CD86 for all cell lines, but the HPV-positive cell line UD-Scc-2.

## Discussion

We tested the hypothesis that HPV-positive HNSCC cells differ in their immune phenotype from HPV-negative ones particularly after treatment with RT in combination with docetaxel that is known, as being a taxane, to have immune stimulatory properties [10].

We revealed for the first time that a single treatment of HNSCC cells with docetaxel does not significantly impact on cell death, release of DAMPs and expression of ICMs. However, combination of docetaxel with hypo-fractionated RT particularly renders HPV-positive HNSCC tumor cells more immunogenic, characterized by increased release of HMGB1 and increased expression of immune stimulatory checkpoint molecules, with hypo-fractionated RT being the main trigger for it.

Cell death analyses revealed that 5 × 3 Gy in combination with docetaxel significantly increased the apoptotic and necrotic cell death rates in all four cell lines, independently of their HPV status. On the other hand, hypo-fractionated RT itself only led to a higher number of apoptotic and necrotic cells in the HPV-negative cell line Cal33 and the HPV-positive cell line UD-Scc-2. Chemotherapy hardly resulted in any significant changes, regarding cell death. However, the combination of RT and docetaxel induced increased apoptosis and necrosis in 3 of the 4 cell lines. Golden et al. showed that taxanes are known to have a radio-sensitizing effect [24]. Our findings suggest that radio- and chemosensitivity of HNSCC cells regarding cell death induction is rather cell line-dependent than associated with the HPV-status. However, generally apoptotic cell death is considered to be immunosuppressive, whereas necrotic cell death is immunostimulatory and therefore the favored outcome in cancer therapy, as it aims for a higher immunogenicity [25,26]. Bearing that in mind, comparing apoptosis and necrosis rate, the HPV-negative cell lines had a higher apoptosis rate. In contrast, irradiating HPV-positive cell lines resulted in a higher necrotic cell death rate, which was also recently shown by Wimmer et al. [13]. Thus indicating, that the HPV-negative cell lines killed by RT alone or in combination with docetaxel could rather suppress immune response, whereas the treated HPV-positive cell lines would stimulate an immune response, supporting better outcome of HPV-positive associated head and neck tumors. But one has to mention that apoptotic cells can also be immunogenic, thereby contribution to local and systemic radio(chemo)therapy-induced anti-tumor immune responses [27,28]. To draw final conclusions, future *in vivo* testing has to be performed according the guidelines for immunogenic cancer cell death [29].

When looking at extracellular HSP70 released by dying tumor cells, acting as DAMPs and resulting in a pro-inflammatory response [6,30,31], we found the increased release of HSP70 to be treatment dependent for all cell lines and independent on the HPV status. RCT resulted in a significantly higher HSP70 concentration for all cell lines but UD-Scc-2, where nonetheless a tendency of an augmentation could be seen. HSP70 promotes immune system activation by facilitating antigen presentation of DCs, as well as the consecutive activation of CD8^+^ cytotoxic T cells (CTLs). Moreover, direct activation of natural killer (NK) cells by DAMPs is possible [32]. HMGB1, while being dependent on Toll-like receptor 4 (TLR4), also improves antigen cross-presentation by DCs, hence leading to an adaptive immune response against the tumor [33,34]. An increased expression of HMGB1 following RCT correlates with higher overall survival and decreased tumor recurrence [35,36].

Kowalczyk et al. found that radiation or CT with cisplatin significantly enhanced CTL-mediated HPV-associated target cell lysis [11]. We revealed that only HPV-positive HNSCC cells release significant more HMGB1 in comparison to HPV-negative ones following RT plus docetaxcel. This might also indicate a consecutive better CTL response, what has however to be proven in future experiments. Clasen et al. found that patients with specific T cell response had significantly increased levels of plasma HMGB1 [35,36], hence supporting a better outcome of HPV-positive associated tumors, which might be because of an increased tumor specific T cell response.

Current treatment concepts for HNSCC additionally comprise immune therapy with immune checkpoint inhibitors particularly targeting the PD-1/PD-L1 axis [37]. Even though studies have shown that the infiltration of cytotoxic T cells is far higher in an HPV-positive tumor micro-environments [38,39], there are still large amounts of patients, that do not seem to respond to immunotherapies targeting PD-1/PD-L1. Therefore, it is pivotal to optimize existing immunotherapies, but also gain further knowledge about the expression of other targetable ICMs [40,41]. We found that, for all examined cell lines both hypo-fractionated RT with 5 × 3 Gy and the combination with docetaxel led to a significant increase in most of the examined immune suppressive ICMs. On the other hand, exclusive docetaxel did not cause any significant changes. Thus, we conclude that the augmented expression is mainly caused by the hypo-fractionated RT and not by the CT, independently of the HPV-status, which was also stated by recent findings [13]. Anti-PD-1 immune checkpoint inhibitors, such as nivolumab or pembrolizumab, are already included in therapy for unresectable or metastatic HNSCC [42]. Nevertheless, the ICM herpes virus entry molecule (HVEM) could also be a promising target, as we identified it to be significantly upregulated following RCT of HNSCC. HVEM as a potential target for immune checkpoint inhibitors has already been stated by other studies, here regarding melanoma, prostate and lung cancer [43], [44], [45].

When looking at immune stimulatory immune checkpoint molecules, we found for the first time that hypo-fractionated RT with 5 × 3 Gy in combination with docetaxel had the biggest impact on their upregulation. In contrast to the immune suppressive ICMs, we detect here a dependence on the HPV-status particularly for one of the examined ICMs, CD137-L. CD137 is a member of the tumor necrosis receptor superfamily and considered to be a co-stimulatory molecule resulting in activation and survival in CD8^+^ T cells. It is an inducible cell surface receptor that is mainly found on activated T cells. It is involved in differentiation and survival signaling in T cells upon binding of its natural partner CD137-L. Lucido et al showed that HNSCC tumor clearance is further potentiated by local tumor cell expression of CD137L [46]. Our analyses revealed that the expression of CD137-L was upregulated following 5 × 3 Gy plus docetaxel by the HPV-positive cell lines UD-Scc-2 and UM-Scc-47. CD137 agonist were found to induce DC-maturation and tumor antigen cross-presentation [47], indicating that higher CD137-L expression after RCT on HPV-associated tumor cells should be beneficial [48]. Besides CD137-L, also CD70, ICOS-L and OX40-L were significantly increased on HPV-positive HNSCC cells after RCT, but also partly by HPV-negative ones. This suggests that targeting these immune stimulatory ICMs in combination with RT and CT in HNSCC might further improve therapy efficacy in the future. Recent clinical trials suggest that distinct combinations of RT with immune therapies targeting immune checkpoint molecules in head and neck squamous cell carcinoma induce beneficial anti-tumor immune responses [49]. Our preclinical results are in line that individual analyses of immune alterations in HNSCC after RCT should be performed to improve personalized radio-immunotherapies for HNSCC. Alternatively, toxic chemotherapy might be replaced by targeting specific ICMs in combination with RT, as already examined in clinical trials [50].

To initiate CTL-mediated anti-tumor immune responses, DC maturation is key as initial step, as immature DCs are ineffective Ag presenting cells and T cell stimulators [51]. Several factors play a role in the process of DC maturation. A major source of such factors is ICD, which includes alterations in cell surface configurations and the release of several soluble mediators such as HMGB1 [52]. Moreover, HSP70, also released by necrotic cells, binds on TLR-4 on DCs and lead to a maturation induction [53,54]. In our *ex vivo* experiment with hmDCs, the maturation cocktail significantly increased the surface expression of activation markers on these DCs, proofing the functionality of the differentiated DCs. However, although cell death and the release of HSP70 and HMGB1 was induced by RT in combination with docetaxel, we could not detect an increase of activation markers on hmDCs after co-culture with the tumor cells. Consequently, we conclude that the increased expression of stimulatory ICMs and the increased release of DAMPs were not sufficient to activate the hmDCs in our chosen setting. This might be due to lack of a lymph node setting being present *in vivo* with collective behavior of the immune cells [55]. Recent clinical trials testing combination of RCT with immune therapy were not as positive as hypothesized. This might also be due to irradiation of lymph nodes in the head and neck region that dampens RT-induced anti-tumor immune responses [56]. *In vivo* experiments are required in the future, as the treated tumor cells could also be taken up by other DC subsets, such as cDC1s, which are specialized to cross-present tumor antigen to CD8+ T cells [57]. Further, the immune stimulatory properties of particularly HPV-positive HNSCC cells might play a key role not in the priming/initiation phase of an anti-tumor immune response, but rather in the effector phase by (re-)activating T cells.

## Conclusion

With our experimental setting we have shown for the first time that the DAMP HMGB1 is only released from HPV-positive HNSCC tumor cells after fractionated irradiation and that this can be significantly enhanced with taxane-based chemotherapy. Together with the HPV-associated upregulation of ICOS-L and CD137-L following RCT, this provides another hint why HPV-positive HNSCC seem to be more immunogenic and thus have a better prognosis. Finally, we identified other than PD-1/PD-L1 axis related ICMs in HNSCC, such as HVEM, that are upregulated after RCT and could be envisaged as individual targets in multimodal therapies for HNSCC in the future.

## Funding

This research was funded by the Interdsiciplinary Center for Clinical Research Erlangen (IZKF Erlangen).

## Data availability statement

The data presented in this study are available on reasonable request from the corresponding author.

## CRediT authorship contribution statement

**Fridolin Grottker:** Methodology, Data curation, Visualization, Funding acquisition. **Simon Gehre:** Methodology, Data curation, Writing – original draft. **Clara M. Reichardt:** Methodology, Data curation. **Azzaya Sengedorj:** Methodology, Visualization. **Tina Jost:** Methodology, Visualization. **Thorsten Rieckmann:** Writing – review & editing. **Markus Hecht:** Validation, Writing – review & editing. **Antoniu-Oreste Gostian:** Conceptualization, Validation, Writing – review & editing. **Benjamin Frey:** Conceptualization, Writing – review & editing, Supervision. **Rainer Fietkau:** Validation, Writing – review & editing. **Udo S. Gaipl:** Conceptualization, Validation, Writing – original draft, Writing – review & editing, Supervision, Project administration, Funding acquisition. **Michael Rückert:** Conceptualization, Validation, Data curation, Writing – original draft, Writing – review & editing, Supervision, Project administration.

## Declaration of Competing Interest

The authors declare no relevant conflict of interest regarding this manuscript.
